# Chicago Veterinary College

**Published:** 1900-08

**Authors:** 


### COMMENCEMENT EXERCISES.

### CHICAGO VETERINARY COLLEGE.

THE seventeenth annual commencement exercises were held on Tuesday, March 27th, at the College Auditorium. Numbers of friends of the graduating class were present. Prof. Joseph Hughes presided. The degree of M.D.C. was conferred upon the following gentlemen: A. W. Beveridge, Belle Centre, O.; T. W. Corkery, Urbana, Ill.; F. H. Davis, Ottawa, Ill.; H. A. Hela, Cokato, Minn.; J. J. Heldring, Amsterdam, Holland; J. Hill, Jr., North Platte, Neb.; H. Jensen, Ph.G., Weeping Water, Neb.; O. H. Leonhart, New Washington, O.; L. F. Miller, La Salle, Ill.; A. J. Roll, V.S., Natrona, Pa.; G. Sprenger, Hastings, Neb.; L. C. Tasche, Sheboygan, Wis.; A. M. Wray, Richmond, Ill.

Dr. H. Jensen, of Weeping Water, Neb., was awarded the gold meadal for the best general average in all branches. Dr. H. A. Hela, of Cokato, Minn., received the prize for Theory and Practice, consisting of a $25 case of instruments. Dr. F. H. Davis, of Ottawa, Ill., was presented with $25 worth of books on account of his standing highest in anatomy.

In conclusion, Prof. A. H. Baker made a few interesting remarks concerning the future prospects of the veterinarian, and wished the parting graduates a prosperous career.

